# Intra- versus retroplacental hematomas: a retrospective case-control study on pregnancy outcomes

**DOI:** 10.1186/s12884-017-1539-6

**Published:** 2017-10-26

**Authors:** Johannes Ott, Philipp Pecnik, Regina Promberger, Sophie Pils, Julia Binder, Kinga M. Chalubinski

**Affiliations:** 1 Clinical Division of Gynecological Endocrinology and Reproductive Medicine, Medical University of Vienna, Waehringer Guertel 18-20, 1090 Vienna, Vienna Austria; 2 Department of Obstetrics and Fetomaternal Medicine, Medical University of Vienna, Waehringer Guertel 18-20, 1090 Vienna, Vienna Austria; 3 Second Department of Internal Medicine, Klinikum Wels-Grieskirchen, Wagnleithnerstraße 27, 4710 Grieskirchen, Upper Austria Austria; 4 Department of Obstetrics and Gynecology, Saint John of God Hospital Eisenstadt, Johannes-von-Gott Platz 1, 7000 Eisenstadt, Burgenland Austria

**Keywords:** Intraplacental hematoma, Retroplacental hematoma, Intrauterine fetal death, Placental insufficiency, Pregnancy complications

## Abstract

**Background:**

Intrauterine hematomas are a common pregnancy complication. The literature lacks studies about outcomes based on hematoma localization. Thus, we aimed to compare pregnancies complicated by an intraplacental hematoma to cases with a retroplacental hematoma and to a control group.

**Methods:**

In a retrospective case-control study, 32 women with an intraplacental hematoma, 199 women with a retroplacental hematoma, and a control group consisting of 113 age-matched women with no signs of placental abnormalities were included. Main outcome measures were pregnancy complications.

**Results:**

Second-trimester miscarriage was most common in the intraplacental hematoma group (9.4%), followed by women with a retroplacental hematoma (4.2%), and controls (0%; *p = 0.007*). The intraplacental hematoma group revealed the highest rates for placental insufficiency, intrauterine growth retardation, premature preterm rupture of membranes, preterm labor, preterm delivery <37 weeks, and early preterm delivery <34 weeks (*p < 0.05*), followed by the retroplacental hematoma group. When tested in multivariate models, intraplacental hematomas were independent predictors for placental insufficiency (*ß = 4.19, p < 0.001*) and intrauterine growth restriction (*ß = 1.44, p = 0.035*). Intrauterine fetal deaths occurred only in women with a retroplacental hematoma (*p = 0.042*).

**Conclusions:**

Intra- and retroplacental hematomas have different risk profiles for the affected pregnancy and act as independent risk factors.

## Background

Intrauterine hematomas are a common pregnancy complication, and can occur at any time during the entire pregnancy, with an associated appearance of obstetrical bleeding in 5–25% in the first trimester, putting mother and child at risk [1]. The incidence of hematomas in the first trimester is reportedly 4–22%, with smaller hematomas often occurring in the first trimester, whereas larger intrauterine masses are more common in the second trimester [2]. Studies and reviews have shown that both early and late hematomas are associated with a higher rate of adverse events, such as vaginal hemorrhage, miscarriage, early delivery, pregnancy-induced hypertension, pre-eclampsia, gestational diabetes, intrauterine growth restriction, and even stillbirth [2–5]. The term “intrauterine hematoma” encompasses several entities that commonly include retroplacental, subchorionic, and subamniotic hematomas. As retroplacental and marginal hematomas are most frequent, most existing studies about the outcome of pregnancies with hematomas refer to these two entities, with intraplacental hematomas sparsely evaluated.

Placental bleedings can be classified according to their as retroplacental, subchorionic, subamniotic, or intraplacental hematomas. The latter are rare and the literature on this entity is scarce. Notably, in a recent histopathological study, rounded intraplacental hematomas revealed morphological features than other than parabasally located intervillous thrombohematomas [6]. Sonographically, intraplacental hematomas are located in the intervillous cavity of the placenta, whereas retroplacental hematomes are located between the basal plate and myometrium, lifting the placental parenchyma toward the amniotic cavity [7]. This suggests that intraplacental hematomas are a separate entity, and validates our clinical experience that intraplacental hematomas are associated with an extraordinarily increased risk of fetal and maternal adverse events. Thus, we believe that this risk exceeds that of the other types of intrauterine hematomas. To date, the literature focused on intrauterine hematomas is general in nature, and no studies have evaluated the outcomes of intraplacental hematomas separately. Thus, we aimed to evaluate neonatal and obstetric outcomes in all patients with an intraplacental hematoma who had been diagnosed between 2006 and 2012 at the Department of Obstetrics and Fetomaternal Medicine of the Medical University of Vienna, Austria. These 32 cases were compared to (i) women who suffered from a retroplacental hematoma, and (ii) an aged-matched control group that included women with no signs of placental abnormalities.

## Methods

### Patient population and study design

In a retrospective study, all women with an intraplacental hematoma (*n* = 32) and a retroplacental hematoma (*n* = 199) who had delivered at the Department of Obstetrics and Fetomaternal Medicine of the Medical University of Vienna, Austria, between August 2006 and January 2012, were identified. As a control group, 113 age-matched women with no signs of placental abnormalities who had also delivered at our department within the same time period were also included. For selection of controls, a large database including all deliveries at the department was used and the procedure was performed by “case-control-matching” in SPSS 17.0 software (SPSS Inc., 1989–2009). Retrospective chart review was performed using the PIA Fetal Database software (GE-Viewpoint, Wessling, Germany).

**Fig. 1:**
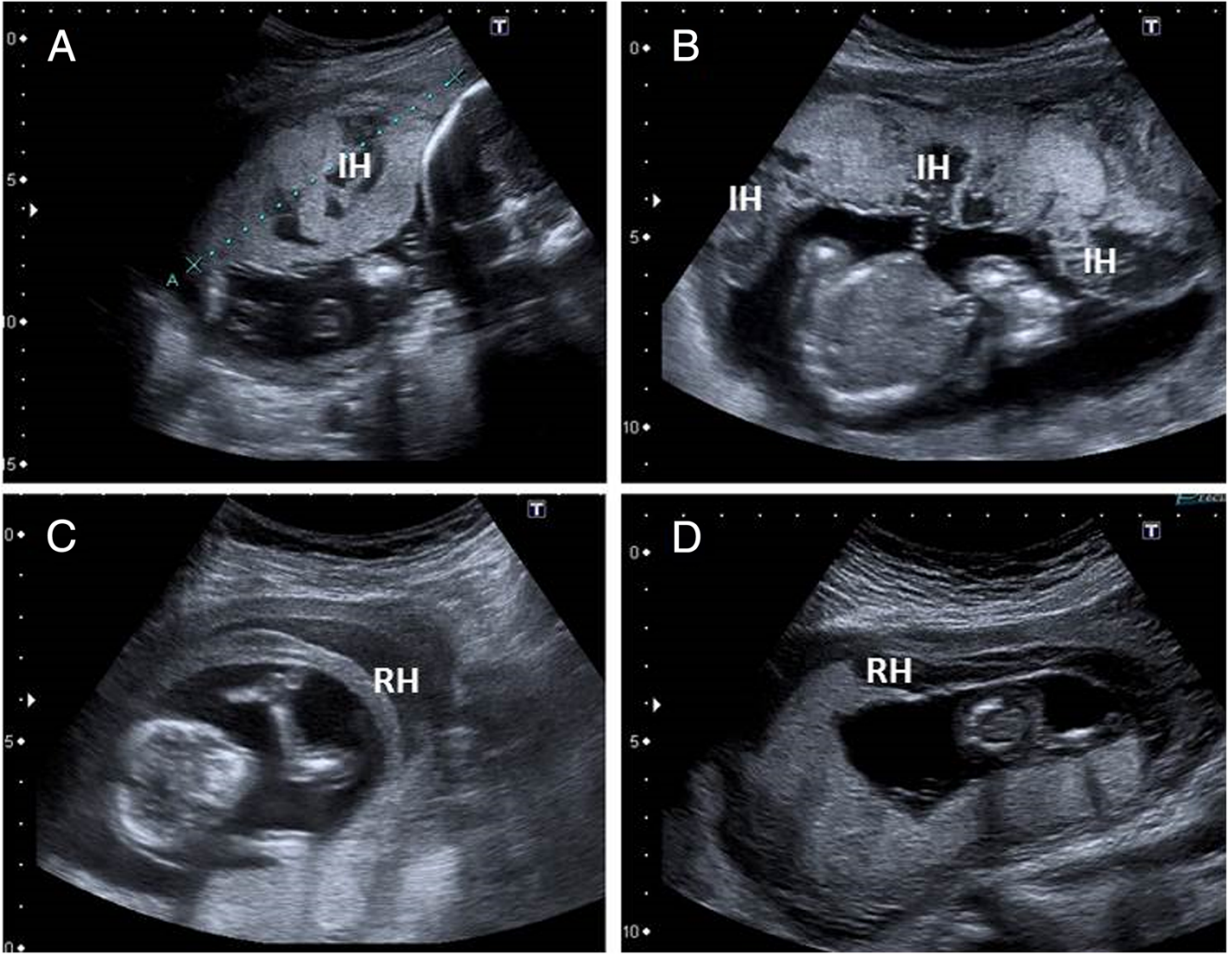
Sonographic appearance of intraplacental hematomas (“IH”, **a** and **b**) and retroplacental hematomas (“RH”, **c** and **d**)

All hematomas were diagnosed during pregnancy via ultrasound in the course of either a routine examination (first or second trimester screening) or an examination for vaginal bleeding. All hematoma-specific ultrasound examinations were performed by one highly experienced operator (K.M.C.), using commercially available, real-time equipment. In detail, a Toshiba Power Vision (Toshiba, Tokyo, Japan) ultrasound machine was used until 2007, and a Toshiba Aplio MX machine from 2007 to 2012. For each patient, the whole placenta was scanned in a systematic fashion, using both gray-scale ultrasound and color-flow mapping. Standard 3.75 MHz linear or sector transducers were used for abdominal ultrasound and a 7.5 MHz transducer was used transvaginally. Doppler power settings were at the level approved for fetal use. The diagnosis of an intrauterine hematoma was established during either routine first- and second-trimester screening or during a non-routine ultrasound for vaginal bleeding. Intraplacental hematomas were defined as sonographically diagnosed hypoechoic masses, with no signs of blood flow, located in the intervillous cavity of the placenta (Fig. 1a and b). In the present analysis, this entity also included massive subchorionic hematomas [7]. Retroplacental hematomas were defined as a hypoechoic area between the basal plate and myometrium, lifting the placental parenchyma toward the amniotic cavity (Fig. 1c and d) [7].

In patients of the control group, intrauterine hematomas had been excluded during first- and second-trimester screenings, as well as in the course of the final ultrasound before delivery, i.e., after the onset of spontaneous labor or 1 day before elective Cesarean section.

Patients with an intrauterine hematoma underwent regular follow-up examinations, including gray-scale and Doppler sonography on an individual basis, at least every 2 weeks. The study was approved by the Institutional Review Board of the Medical University of Vienna (IRB number: 1681/2014). Neither written nor verbal informed consent is unnecessary in retrospective studies according to the Ethics Committee of the Medical University of Vienna and was, thus, not obtained.

### Parameters analyzed

As outcome parameters, we focused on the incidences of the following pregnancy complications: pregnancy-induced hypertension, defined as a blood pressure of 140 mmHg systolic or higher or 90 mmHg diastolic or higher that occurred after 20 weeks of gestation in a woman with previously normal blood pressure with no proteinuria or other signs of organ dysfunction [8]; preeclampsia, defined as blood pressure of 140 mmHg systolic or higher or 90 mmHg diastolic or higher that occurred after 20 weeks of gestation in a woman with previously normal blood pressure, and with proteinuria, defined as urinary excretion of 0.3 g protein or higher in a 24-h urine specimen [9]; placental insufficiency, characterized by the presence of fetal growth restriction, reduced amniotic fluid, and impaired fetal oxygenation and which demonstrated pathological Doppler indices in the umbilical (UA) and uterine arteries [10]. Reference ranges for Doppler parameters were based on the observations of Schaffer et al. [11]; intrauterine growth retardation, defined as an abdominal circumference < 5th percentile, measured at the first visit during the second or early third trimester [12]; intrauterine fetal death; preterm premature rupture of membranes, defined as spontaneous rupture of the membranes prior to the onset of labor before 37 gestational weeks [13]; preterm labor, defined as regular uterine contractions leading to cervical dilation, effacement, or both, or initial presentation with regular contractions and cervical dilation of at least 2 cm between 20 + 0 weeks of gestation and 36 + 6 weeks of gestation preterm delivery <37 weeks [14]; early preterm delivery < 34 + 0 weeks; and complete placental abruption which was defined as a complete separation of the placental lining from the uterus (in contrast to a retroplacental hematoma). In addition, we included mother’s age, parity, the maximum diameter of the hematoma (divided into hematomas < 5 cm and ≥ 5 cm) and whether the woman had suffered from previous intrauterine fetal death (IUFD) as parameters in the predictive models [5].

### Statistical analyses

Nominal variables are reported as numbers and frequencies, and continuous variables with median and interquartile ranges. Differences between groups were tested using ANOVA or Welsch tests, where appropriate, for numeric variables and Chi square or Fisher’s exact test for categorical variables. To assess the predictive factors for placental insufficiency and intrauterine growth retardation, multivariate logistic regression models were used. Coefficient estimates, β and standard error se(β), as well as corresponding *p*-values, are given for these analyses. Statistical analysis was performed using the SPSS 17.0 software (SPSS Inc., 1989–2009). Differences were considered statistically significant if *p < 0.05*.

## Results

There were no missing data. Basic patient characteristics are shown in Table 1. Notably, the three groups differed in terms of the following parameters (*p < 0.05*). The previous incidence of intrauterine fetal death was highest among women with an intraplacental hematoma (12.5%), followed by those with a retroplacental hematoma (2.5%). Chorionic villous sampling preceded an intrauterine hematoma in 6.3% (*n* = 2) compared to none in the retroplacental hematoma group. Retroplacental hematomas were diagnosed significantly earlier (median 13 weeks; interquartile range, 12–21) than intraplacental hematomas (median 24 weeks; interquartile range, 22–29; *p < 0.001*).

**Table 1:** Patient characteristics

	Intraplacental hematoma (*n* = 32)	Retroplacental hematoma (*n* = 119)	Controls (*n* = 113)	*p*
Age (years)^a^	31 (27;35)	31 (26;34)	31 (27;36)	0.394
Parity (n)^a^	1 (0;2)	2 (0;2)	2 (0;2)	0.486
Previous PIH^b^	0	3 (2.5)	0	0.348
Previous IUGR^b^	1 (3.1)	1 (0.8)	6 (5.3)	0.138
Previous preeclampsia^b^	1 (3.1)	3 (2.5)	1 (0.9)	0.534
Previous IGDM^b^	1 (3.1)	4 (3.4)	2 (1.8)	0.653
Previous preterm delivery^b^	3 (9.4)	4 (3.4)	7 (6.2)	0.287
Previous IUFD^b^	4 (12.5)	3 (2.5)	0	*0.001*
Chorionic villous sampling^b^	2 (6.3)	0	1 (0.9)	*0.039*
IGDM^b^	3 (9.4)	22 (18.5)	26 (23.0)	0.219
Gestational age at diagnosis (completed weeks)^a^	24 (22–29)	13 (12–21)	–	*<0.001*

Data are provided as ^a^median (interquartile range) or ^b^n (%)

*Abbreviations*: *PIH* pregnancy induced hypertension, *IUGR* intrauterine growth restriction, *IGDM* insulin-dependent gestational diabetes mellitus, *IUFD* intrauterine fetal death

Maximum hematoma diameter was as follows: a hematoma exceeding 5 cm of maximum diameter was found in 14/29 intraplacental cases (48.3%) compared to 33/114 (28.9%) in retroplacental cases (*p = 0.041*).

In the intraplacental hematoma, retroplacental hematoma, and control groups, ten (31.3%), 71 (59.7%), and 4 (3.5%) women, respectively, suffered from vaginal bleeding at least once during the course of their pregnancy (*p < 0.001*). The incidence of second-trimester miscarriage was highest in the intraplacental hematoma group (*n* = 3, 9.4%), followed by women with a retroplacental hematoma (*n* = 5, 4.2%) and controls (*n* = 0; *p = 0.007*).

The latter patients were excluded from the subsequent analyses on pregnancy outcomes, since data on the evaluated outcome parameters were available only from week 24 + 0 onwards. In short, the intraplacental hematoma group (*p < 0.05*) revealed significantly higher rates of placental insufficiency, intrauterine growth retardation, premature preterm rupture of membranes, preterm labor, preterm delivery < 37 weeks, and early preterm delivery < 34 weeks (Table 2). These findings were associated with differences in gestational age at delivery, birth weight, and rates of Cesarean section between the groups (*p* < 0.001). IUFD occurred only in women with retroplacental hematoma. None of the affected fetuses revealed any birth defects. Median gestational age at diagnosis of IUFD was 24 completed weeks (IQR 23–25). For all of these outcome parameters, women with retroplacental hematoma were still at increased risk compared to the controls. However, intrauterine fetal deaths occurred only in the retroplacental hematoma group (*p = 0.042*).

**Table 2:** Pregnancy outcomes. Patients with second-trimester miscarriage were excluded from these analyses

	Intraplacental hematoma (*n* = 29)	Retroplacental hematoma (*n* = 114)	Controls (*n* = 113)	*p*
Pregnancy-induced hypertension^b^	4 (13.8)	8 (7.0)	12 (10.6)	0.377
Preeclampsia^b^	2 (6.9)	3 (2.6)	9 (8.0)	0.171
Placental insufficiency^b^	9 (31.0)	11 (9.6)	1 (0.9)	*<0.001*
Intrauterine growth retardation^b^	8 (27.6)	14 (12.3)	10 (8.8)	*0.034*
Intrauterine fetal death^b^	0	6 (5.3)	0	*0.042*
Preterm premature rupture of membranes^b^	2 (6.9)	29 (25.4)	14 (12.4)	0.012
Preterm labor^b^	8 (27.6)	26 (22.8)	6 (5.3)	*<0.001*
Preterm delivery^b^	15 (51.7)	34 (29.8)	11 (9.7)	*<0.001*
Early preterm delivery <34 + 0 weeks^b^	10 (31.3)	17 (14.3)	3 (2.7)	*<0.001*
Placental abruption^b^	1 (3.4)	3 (2.6)	1 (0.9)	0.522
Birth weight (g)^a^	2520 (1076;3215)	3020 (2420;3430)	3200 (2870;3545)	<0.001
Gestational age at delivery (completed weeks)^a^	37 (31;39)	39 (36;41)	39 (39;40)	<0.001
Delivery by Cesarean section^b^	18 (69.2)	56 (49.1)	27 (23.9)	<0.001

Data are provided as ^a^median (interquartile range) or ^b^n (%)

In a next step, we tested several parameters for the prediction of placental insufficiency and intrauterine growth restriction (Table 3). For both outcome parameters, retroplacental hematomas significantly increased the risk for the development of the complication. The presence of a retroplacental hematoma outweighed all other tested parameters apart from previous IUGR as a predictor of its recurrence. When performing a similar analysis for early preterm delivery < 34 + 0 weeks (Table 4), both intra- and retroplacental hematomas, lower parity and presence of IUGR increased the risk significantly (women with IUFD excluded). When taking intra- and retroplacental locations together, the presence of hematoma was also associated with a significantly increased risk for early preterm delivery (ß = 2.00 ± 0.63, *p* = 0.001).

**Table 3:** Multivariate logistic regression models for the prediction of placental insufficiency and intrauterine growth retardation

Parameter		Placental insufficiency		ß (se ß)	*p*	IUGR		ß (SD ß)	*p*
		Yes (*n* = 21)	No (*n* = 235)			Yes (*n* = 32)	No (*n* = 224)		
Group^b^	Controls	1 (4.8)	112 (46.1)	reference	0.001	10 (31.3)		reference	0.016
	Intrapl. hematoma	9 (42.9)	20 (8.2)	4.20 (1.17)		8 (25.0)		1.81 (0.63)	
	Retropl. hematoma	11 (52.4)	103 (42.4)	2.93 (1.11)		14 (43.8)		0,94 (0.52)	
Parity (n)^a^		1 (0;2)	2 (0;2)	−0.45 (0.30)	0.133	1 (0;2)	2 (0;2)	--0.45 (0.24)	0.065
Age (years)^a^		32 (26;38)	31 (26;35)	0.08 (0.04)	0.069	30 (23;34)	31 (27;35)	−0.02 (0.04)	0.660
IGDM^b^		3 (14.3)	48 (20.4)	−0.22 (0.72)	0.755	6 (18.8)	45 (20.1)	−0.18 (0.58)	0.759
PIH^b^		4 (19.0)	20 (8.2)	−0.41 (0.98)	0.676	4 (12.5)	20 (8.9)	−0.25 (0.85)	0.772
Preeclampsia^b^		3 (14.3)	11 (4.5)	2.21 (1.17)	0.059	3 (9.4)	11 (4.9)	0.55 (0.99)	0.582
Previous IUGR^b^		0	8 (3.4)	−17.90 (12,437.91)	0.999	6 (18.8)	2 (0.9)	4.02 (0.99)	<0.001
Previous IUFD^b^		2 (9.5)	5 (2.1)	0.76 (1.00)	0.448	1 (3.1)	6 (2.7)	−0.02 (1.16)	0.986
Constant		–	–	−7.14 (1.85)	0.000	–	–	−1.93 (1.11)	0.082

Patients with second-trimester miscarriage were excluded from these analyses

Data are provided as ^a^median (interquartile range) or ^b^n (%)

*Abbreviations*: *Intrapl.* intraplacental, *Retropl.* retroplacental, *IUGR* intrauterine growth retardation, *IGDM* insulin-dependent gestational diabetes mellitus, *PIH* pregnancy-induced hypertension, *IUFD* intrauterine fetal death

**Table 4:** Multivariate logistic regression models for the prediction of early preterm delivery <34 + 0 weeks

Parameter		Early preterm delivery		ß (se ß)	*p*
		Yes (*n* = 26)	No (*n* = 224)		
Group^b^	Controls	3 (11.5)	110 (49.1)	reference	0.001
	Intrapl. hematoma	10 (38.5)	19 (8.5)	2.92 (0.78)	
	Retropl. hematoma	13 (50.0)	95 (42.4)	1.81 (0.70)	
Parity (n)^a^		1 (0;2)	2 (0;2)	−0.63 (0.27)	0.021
Age (years)^a^		31 (26;35)	31 (27;35)	0.04 (0.03)	0.283
IGDM^b^		2 (7.7)	47 (21.0)	−0.98 (0.82)	0.377
PIH^b^		3 (11.5)	21 (9.4)	−1.45 (1.07)	0.235
Preeclampsia^b^		3 (11.5)	11 (4.9)	1.81 (1.12)	0.105
IUGR^b^		8 (30.8)	23 (10.3)	1.27 (0.56)	0.023
Previous IUGR^b^		0	8 (3.6)	−19.06 (12,628.30)	0.999
Previous IUFD^b^		2 (7.7)	5 (2.2)	0.71 (1.01)	0.486
Constant		–	–	−4.40 (1.39)	0.001

Patients with second-trimester miscarriage and IUFD were excluded from this analysis

Data are provided as ^a^median (interquartile range) or ^b^n (%)

*Abbreviations*: *Intrapl.* intraplacental, *Retropl.* retroplacental, *IUGR* intrauterine growth retardation, *IGDM* insulin-dependent gestational diabetes mellitus, *PIH* pregnancy-induced hypertension, *IUFD* intrauterine fetal death

Then, the impact of gestational age at hematoma diagnosis on pregnancy outcome was assessed. Women with placental insufficiency revealed a higher median gestational age at diagnosis (23 completed weeks, IQR 15–27) than those without (15 weeks, IQR 12–24; *p* = 0.034), whereas there was no difference between women with and without IUGR (median 21 weeks, IQR 12–24, versus median 15, IQR 12–25, respectively; *p* = 0.851). Median gestational age at diagnosis was higher in women with early preterm delivery (23 weeks, IQR 14–27) than in patients who delivered after 34 + 0 weeks (14, IQR 12–24; *p* = 0.038). When dividing women into those with a first-trimester diagnosis of hematoma (*n* = 70) versus those with a second-trimester diagnosis (*n* = 73), placental insufficiency was more frequent after a second- (15/73, 20.5%) than after a first-trimester diagnosis (5/70, 7.1%; *p* = 0.029). The same was found for early preterm delivery (patients with IUFD excluded: 18/71, 25.4% versus 5/66, 7.6%, respectively; *p* = 0.006) but not for IUGR (14/73, 19.2% versus 8/70, 11.4%, respectively; *p* = 0.249).

## Discussion

This retrospective study provided the following key findings: (i) both intra- and retroplacental hematomas were associated with increased incidences of pregnancy-related complications, including placental insufficiency, intrauterine growth retardation, and preterm labor and (early) preterm delivery. These findings are underlined by the differences in birth weight and gestational age at delivery between the groups; (ii) women with an intraplacental hematoma were at an even higher risk for these complications than those with a retroplacental hematoma. In contrast, only the latter type of hematoma was associated with IUFD in our study population; (iii) retroplacental hematomas revealed a significantly earlier onset than intraplacental hematomas; and (iv) intraplacental hematomas were an independent risk factor for the development of placental insufficiency and IUGR.

Undoubtedly, intrauterine hematomas put the affected women at an increased risk for pregnancy-related complications. The question is whether differentiating hematomas according to their exact position would be justified in order to provide adequate information about associated risks. One might argue that the accurate position of a hematoma has only descriptive value, because there were no relevant differences reported in outcome according to a previous publication [1]. However, we are the first to describe intraplacental hematomas as a separate entity in a clinical setting. According to the results of our study, pregnancies complicated by intraplacental hematomas would carry a different risk profile than pregnancies with a retroplacental hematoma. In particular, the risks for placental insufficiency, growth retardation, preterm labor, and (early) preterm delivery were significantly higher for women with intraplacental hematomas. Notably, in multivariate analyses, intraplacental hematomas were an important risk factor for the development of placental insufficiency and growth restriction (Table 3) as well as for early preterm delivery (Table 4). In intraplacental hematomas, most of the blood is reportedly maternal [7]. However, these hematomas likely form a leak in the fetal circulation as well. Thus, fetal anemia, and, consequently, underperfusion might result. Particularly in the case of profound placental bleeding, a massive reduction in placental function can occur [7, 15]. This also fits the observation that intraplacental hematomas were significantly larger than those in a retroplacental location. This should also be considered in the context of the size of retroplacental hematomas. Those retroplacental hematomas large enough to cause adverse prenatal effects on the fetus may also produce secondary abnormalities, including intraplacental bleeding [7]. In this case, patients would have been assigned to the intraplacental hematoma group in our study.

Notably, whether the size of an intrauterine hemorrhage would correlate with a worse pregnancy outcome, and therefore, could be used as a predictive marker, has been previously evaluated. However, the results did not reach significance [3, 16]. This might have been due to the fact that the exact location of the hematoma exact location was not considered.

Preterm labor was quite common in women with intraplacental hematomas (Table 2). It has already been suggested that these bleeding events are accompanied by the release of cytokines [7]. This could trigger preterm labor. However, retro- but not intraplacental hematomas were associated with a high rate of premature preterm rupture of membranes. We consider this to be due to either cytokine release or the fact that retroplacental bleeding, when it extends along the decidua basalis and reaches the placental margin, might lead to local irritation of the amnion.

Notably, cases of IUFD occurred only in the retroplacental hematoma group. We believe that this was due to an “all-or-none” phenomena. Retroplacental hematomas seemed to have developed significantly earlier, which is suggested by the lower gestational age at initial diagnosis (median 13 vs. 24 weeks). In these earlier weeks of gestation, significant impairment of fetal perfusion more likely leads to IUFD. One might also argue that smaller, and, thus, more irrelevant retroplacental hematomas that developed early did not cause symptoms, such as vaginal bleeding, and, consequently, remained undiagnosed. It has already been suggested that many retroplacental hematomas are easily overlooked [7]. This possible bias could also explain the high IUFD rate in the retroplacental hematoma group.

We would like to provide the following hypotheses to explain the fact that intraplacental hematomas obviously developed later in pregnancy than retroplacental hematomas. (i) As suggested previously, subchorionic/retroplacental hemorrhage might be the result of abnormal development of the placental membranes in the first trimester [17]. In contrast, the blood of intraplacental hematomas is mostly maternal and intervillous circulation is not fully established at the end of the first trimester, suggesting that this hematoma entity must develop later in pregnancy [7, 18]. (ii) The source of bleeding could also be linked to trophoblastic activity that leads to extensive expansion of the spiral ateries’ lumina, beginning with the second trimester. Accordingly, the massive intraplacental hematomas could develop only from this gestational age on. Moreover, it has already been mentioned that rounded intraplacental haematomas form as a result of disruption of vasculopathic decidual arterioles in a setting of maternal vascular underperfusion and are thus etiologically distinct from retroplacental hematomas [6].

Thus, if a hematoma is found early in pregnancy, i.e. within the first 21 weeks of gestation, its location will likely be retroplacental, whereas intrauterine hematomas found at a higher gestational age will be intraplacental in the majority of cases. However, gestational age at diagnosis will not predict hematoma location with apodictic certainty, since the presented median gestational ages at diagnosis were associated with quite a big interquartile range. Moreover, it has been mentioned that gestational age at diagnosis might predict pregnancy risks [19, 20]. However, we consider hematoma type of higher predictive value for pregnancy complications than age at diagnosis; women with a second-trimester diagnosis which is associated with the finding of an intraplacental hematoma in the majority of cases carried the highest risks.

The retrospective nature of our study may have introduced some kind of selection bias, as discussed above, and thus, must be considered a study limitation. We cannot provide exact data on changes in sonographic presentation of the hematomas which has to be considered a study limitation. From our clinical experience, hematomas did not get absorbed during follow-up, but their sonographic appearence changed to a more inhomogeneous structure with varying degrees of echogenicity. Neither we are able to provide data about histo−/pathological examinations of the affected placentae. Only a minority of the placentae have been sent for pathological examination, a limitation that is associated with the retrospective study design. Moreover, in case of retroplacental hematomas such an examination seems of only minor impact, since these hematomas are striped off easily. However, we present the first clinical outcome data that could potentially differentiate intra- and retroplacental hematomas.

## Conclusions

Intra- and retroplacental hematomas have different risk profiles for the affected pregnancy. While the rate of IUFD was highest in women with retroplacental hematomas, intraplacental hematomas put the fetus at a significant risk for growth retardation. Clinicians should be aware of these different risk profiles. Prospective studies are warranted to confirm our observations and evaluate adapted treatment strategies.

## Abbreviations

IUFD: Intrauterine fetal death
IUGR: Intrauterine growth restriction
